# IgE receptor responsiveness of basophils in chronic inducible urticaria

**DOI:** 10.3389/fimmu.2022.995596

**Published:** 2022-09-23

**Authors:** Mayuko Mizuno, Yoshiko Oda, Shinya Imamura, Ken Washio, Takeshi Fukumoto, Atsushi Fukunaga

**Affiliations:** ^1^ Division of Dermatology, Department of Internal Related, Kobe University Graduate School of Medicine, Kobe, Japan; ^2^ Department of Dermatology, Division of Medicine for Function and Morphology of Sensory Organs, Faculty of Medicine, Osaka Medical and Pharmaceutical University, Takatsuki, Osaka, Japan

**Keywords:** chronic inducible urticaria, chronic spontaneous urticaria, basophil activation test, anti-IgE-induced histamine release, responsiveness of basophils *via* high-affinity IgE receptor

## Abstract

**Introduction:**

Chronic inducible urticaria (CIndU) is a subgroup of chronic urticaria induced by a specific stimulus. We evaluated basophil characteristics in patients with CIndU and compared with those in patients with chronic spontaneous urticaria (CSU) and healthy controls (HCs).

**Methods:**

Blood was collected from patients, and a basophil activation test (BAT) was performed. Basophil responsiveness and surface marker expression in patients with CIndU were compared with those in patients with CSU and HCs. For some patients with CIndU, blood was collected before and after wheals were induced. In these cases, we compared the responsiveness of basophils before and after the appearance of wheals.

**Result:**

HCs (n=23) and patients with CIndU (n=24) or CSU (n=38) were enrolled in the study. The degree of basophil activation at steady state in patients with CIndU was higher than in HCs. Basophil responsiveness *via* high-affinity IgE receptor (FcϵRI) stimulation with anti-IgE or anti-FcϵRI antibody in patients with CIndU was equivalent to that in HCs, and higher than that in patients with CSU. No abnormalities in IgE and FcϵRI expressions on the surface of basophils in patients with CIndU were observed. When we induced wheals in some patients with CIndU and performed a BAT before and after the appearance of wheals, no significant changes were found.

**Conclusion:**

Peripheral blood basophils in CIndU were slightly activated at steady state, but no abnormalities in basophil responsiveness. In future, a higher number of cases should be enrolled to confirm the role of basophils and refine therapeutic targets for CIndU.

## Introduction

Chronic urticaria is defined as the occurrence of wheals and/or angioedema for more than 6 weeks. Chronic inducible urticaria (CIndU) is a subgroup of chronic urticaria where recurrent pruritic wheals and/or angioedema are induced by a specific stimulus (1). Chronic spontaneous urticaria (CSU) is characterized by the spontaneous appearance of wheals, angioedema, or both and is associated with known (i.e., mast-cell activating autoantibodies) or unknown causes (2) (3) (4). Several studies reported that the responsiveness of basophils *via* the high-affinity IgE receptor (FcϵRI) and IgE pathways was significantly lower in active CSU compared with healthy controls (HCs), with basophil hyporesponsiveness improving during disease remission (5–7). (8) In contrast, anti-IgE-induced histamine release from the basophils of five patients with cold urticaria, a subtype of CIndU, appeared to be equivalent to that of HCs (9). Basophil FcϵRI expression was significantly higher in patients with CSU and CIndU compared with HCs (10, 11). However, there have been few reports on the characteristics of basophils in patients with CIndU. Here, we evaluated the characteristics of basophil in patients with CIndU, including responsiveness and surface marker expression, and compared them with those in patients with CSU.

## Materials and methods

### Study design

An observational study was conducted on patients with CIndU who visited the Dermatological Institute of Kobe University Hospital. Blood was collected from patients diagnosed with CIndU at the time of non-stimulation (when no wheal was present), and a basophil activation test (BAT) was performed. The basophil responsiveness and surface marker expressions of patients with CIndU were compared with those of patients with CSU and HCs. Moreover, in some patients with CIndU, urticaria was induced by a specific physical stimulus in the doctor’s office, and blood was collected before and after wheals were induced. In these cases, we compared the responsiveness of basophils before and after the appearance of wheals.

### Study population

Patients with CIndU and CSU who visited the Department of Dermatology, Kobe University Hospital, agreed to participate in the study, and met the inclusion criteria were enrolled. The study protocol was approved by the Kobe University Institutional Review Board (No. 180186). Inclusion criteria were to be diagnosed with CIndU or CSU by the following items and not to use omalizumab or steroids. Patient with cholinergic urticaria (CholU) are diagnosed by having wheals induced by exercise and/or passive heating (warm bath). Patients with solar urticaria are diagnosed by having wheals induced by exposure to visible and/or ultraviolet light. CSU is diagnosed as wheals that recur for more than 6 weeks without an identifiable cause. HCs were enrolled from healthy adult volunteers without urticaria symptoms and no history of urticaria. Patients treated with omalizumab and oral steroids were excluded at entry. It was set so that no patients were excluded after inclusion.

### Basophil activation test

Whole blood (up to 2 mL) was taken from patients with CIndU, CSU, and HCs using ethylenediaminetetraacetic acid-containing blood collection tubes and assays were performed within 24 hours of blood sampling. An Allergenicity Kit (Beckman Coulter, Fullerton, CA, USA) was used to quantify basophil CD203c expression according to the manufacturer’s instructions (12). The BAT based on CD203c expression was performed as previously described (6) (7). In addition to CD203c, CD63 (H5C6; BioLegend, San Diego, CA) was also analyzed as an activation marker that reflects histamine release (13). Basophil samples were measured by flow cytometry (FACS Verse; BD Biosciences, San Jose, CA). As previously described, the gating technique is shown in the **Supplementary Material** (6) (**Figure S1**). Basophil activation conditions were determined by the mean fluorescence intensity (MFI). CD203c or CD63 expression after anti-IgE (E124-2-8D; Beckman Coulter, Fullerton, CA, USA) or anti-FcϵRI antibody (CRA1; BioAcademia, Osaka, Japan) stimulation was presented as the CD203c or CD63 response ratio, respectively, and used to calculate the responsiveness of basophils. The response ratio was calculated by dividing the stimulation MFI by the baseline MFI. In addition, the results of anti-IgE antibody stimulation were also expressed as the percentage of CD63 positive basophils. The percentage of CD63 positive basophils were determined using a threshold defined as the expression level above which only 5% of basophils in the negative control sample fluoresce, on average.

### Measurement of IgE and FcϵRI levels of basophils

Basophils were incubated with VioBlue-binding, anti-IgE antibody (clone: MB10-5C4) (Miltenyi Biotec, Bergisch Gladbach, Germany), biotinylated anti-FcϵRI antibody (clone: CRA1) (BioAcademia) and APC-Streptavidin (BD Biosciences, Franklin Lakes, NJ) (1.8 mg/mL) which used as a second-step reagent for the anti-FcϵRI antibody and, analyzed by flow cytometry. The measurement of the IgE and FcϵRI levels of basophils and FlowJo analysis were performed as for the BAT after anti-IgE and CRA1 antibody stimulation. IgE and FcϵRI levels were evaluated as the MFI.

### Urticaria control test

The total score for the Urticaria control test (UCT) was determined by the patient (14). The UCT is a simple, validated, four-item questionnaire that can be used for CSU and CIndU to assess the impact of urticaria symptoms on morbidity, quality of life, and quality of treatment over the past four weeks.

### Autologous serum skin test

The autologous serum skin test was performed according to established methods (15). Samples of autologous serum (0.05 mL) were injected intradermally into the volar aspect of the forearm of each subject. The diameters of wheals and erythema were measured after 15 minutes. Reactions were assessed as positive if the diameter of the wheal induced by serum was equal to or larger than 6 mm.

### Statistical analysis

The Kruskal-Wallis test with Dunn’s multiple comparisons test was used for the statistical comparison of three groups with nonparametric variables. The Wilcoxon test was used for the statistical comparison of two groups with nonparametric variables. All statistical analyses were performed using GraphPad Prism 8 (GraphPad Software, San Diego, CA, USA). Two-sided P values <0.05 were considered statistically significant.

## Results

### Study population

Patients with CIndU (n=24) and CSU (n=38), and HCs (n=23) who agreed to participate in this study were enrolled at the Dermatological Institute of Kobe University Hospital (**Table 1**). Patients with CIndU included 7 males and 17 females. The mean age was 40.2 years and the median duration of illness was 10.0 years. The median total serum IgE was 579.5 IU/mL. Patients with CSU included 24 males and 14 females. The mean age was 46.3 years. (**Table 1**). HCs included 7 males and 16 females. The mean age was 35.0 years. CIndU patients included 21 with CholU and 3 with solar urticaria. Five patients with CholU underwent a bathing provocation test and exercise provocation test followed by blood collection (even when wheals were induced) and a BAT. The BAT of these patients was compared before and after the appearance of wheals.

**Table 1:** Clinical and laboratory characteristics of patients with chronic inducible urticaria (CIndU) and chronic spontaneous urticaria (CSU).

Demographics characteristics of patients with CIndU and CSU	CIndU (n=24)	CSU (n=38)	P values
Age, years	40.2 ± 10.3	46.3 ± 16.2	P=0.0239
Female, n (%)	17 (70.8%)	24 (63.1%)	P=0.5339
Disease duration, years	10.0 (1.0-40)	4.0 (0.2-33)	P=0.046
Total IgE (IU/mL)	579.5 (14.2-1275.3)	139.5 (3-4392)	P<0.0001
Basophil count (cell/μL)	68 (18-106)	52.5 (21.4-114)	P=0.9485
UCT	10.7 ± 3.8	7.8 ± 4.1	P=0.0253
ASST positive rate, n (%)	11/16 (68.7%)	7/17 (41.1%)	P=0.1663
Presence of angioedema at baseline, n (%)	7 (29.1%)	1 (2.6%)	P=0.0041
Treatment, n (%)			
H1 antihistamines at the conventional dosage	17 (70.8%)	19 (50%)	P=0.1886
H1 antihistamines at high dosage	5 (20.8%)	14 (36.8%)	P=0.2599
History, n (%)			
Asthma	8 (33.3%)	5 (13.1%)	P=0.1067
Allergic rhinitis	4(16.6%)	2 (5.2%)	P=0.1949
Atopic dermatitis	10 (41.6%)	1 (2.6%)	P=0.0002
Pollinosis	3 (12.5%)	2 (5.2%)	P=0.3459

ASST, Autologous serum skin test; UCT, Urticaria control test.

Data are given as the mean ± standard deviation for age, UCT; n (%) for sex, ASST positive rate, presence of angioedema, treatment, and history; and median (range) for disease duration, serum total IgE, and basophil count.

### Measurement of CD203c, CD63, IgE and FcϵRI levels on basophils at steady state in patients with CIndU, CSU, and HCs

**Figure 1:**
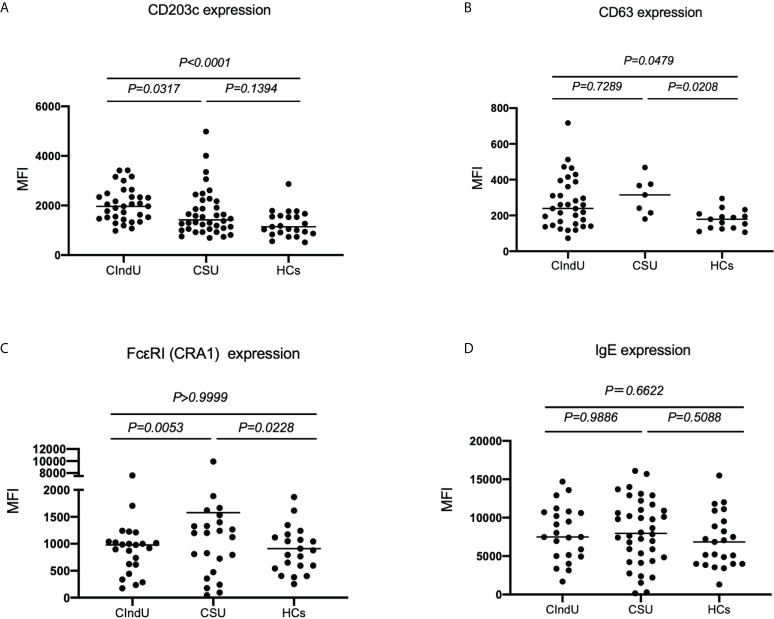
CD203c, CD63, IgE and FcϵRI levels at steady state. **(A)** CD203c expression on basophils, **(B)** CD63 expression on basophils, **(C)** FcϵRI expression on basophils and **(D)** IgE expression on basophils at steady state. Statistical analysis was carried out using the Kruskal-Wallis test with Dunn’s multiple comparisons test.

First, we examined CD203c, CD63, FcϵRI and IgE expression levels on basophils at steady state in patients with CIndU, CSU, and HCs. The expression of CD203c on basophils in patients with CIndU was significantly higher compared with CSU and HCs (**Figure 1A**). The expression of CD63 on basophils in patients with CIndU was significantly higher compared with HCs and was comparable with CSU (**Figure 1B**). The expression of FcϵRI on basophils in patients with CIndU was comparable with HCs and was significantly lower than that in CSU (**Figure 1C**). There were no significant differences in the levels of cell-bound IgE among these three groups (**Figure 1D**).

### Measurement of CD203c and CD63 expressions after anti-IgE or FcϵRI stimulation of basophils in patients with CIndU, CSU, and HCs

**Figure 2:**
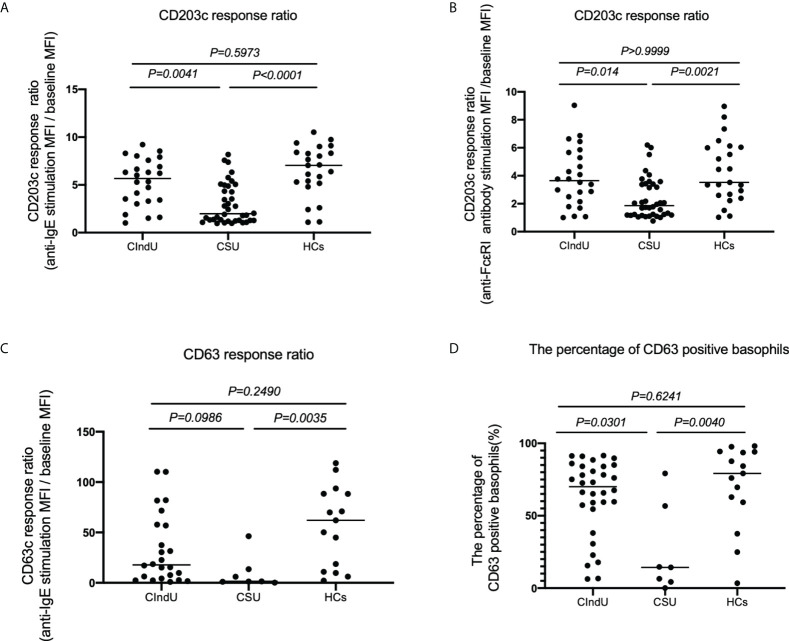
CD203c response ratio, CD63 response ratio and the percentage of CD63 positive basophils. CD203c response ratio of basophils when stimulated with **(A)** anti-IgE or **(B)** anti-FcϵRI antibody. **(C)** CD63 response ratio of basophils when stimulated with anti-IgE, and **(D)** the percentage of CD63 positive basophils when stimulated with anti-IgE. Statistical analysis was carried out using the Kruskal-Wallis test with Dunn’s multiple comparisons test.

Next, we analyzed the expressions of the activation markers CD203c and CD63 with anti-IgE or FcϵRI stimulation in patients with CIndU, CSU, and HCs to examine basophil reactivity *via* FcϵRI. When peripheral blood basophils were stimulated with anti-IgE antibody, the upregulation of CD203c expression on basophils in patients with CIndU was comparable with HCs and was significantly higher than that in CSU (**Figure 2A**). When peripheral blood basophils were stimulated with anti-FcϵRI, the upregulation of CD203c expression on basophils in patients with CIndU was comparable with HCs and was significantly higher than that in CSU (**Figure 2B**). When peripheral blood basophils were stimulated with anti-IgE antibody, similar results were obtained when the detection activation marker was also CD63 (**Figure 2C**). The percentage of CD63 positive basophil also showed similar results when peripheral blood basophils are stimulated with anti-IgE antibody (**Figure 2D**).

### Measurement of CD203c, CD63, IgE and FcϵRI levels on basophils at steady state in patients with CholU as a subgroup of CIndU before and after the appearance of wheals

**Figure 3:**
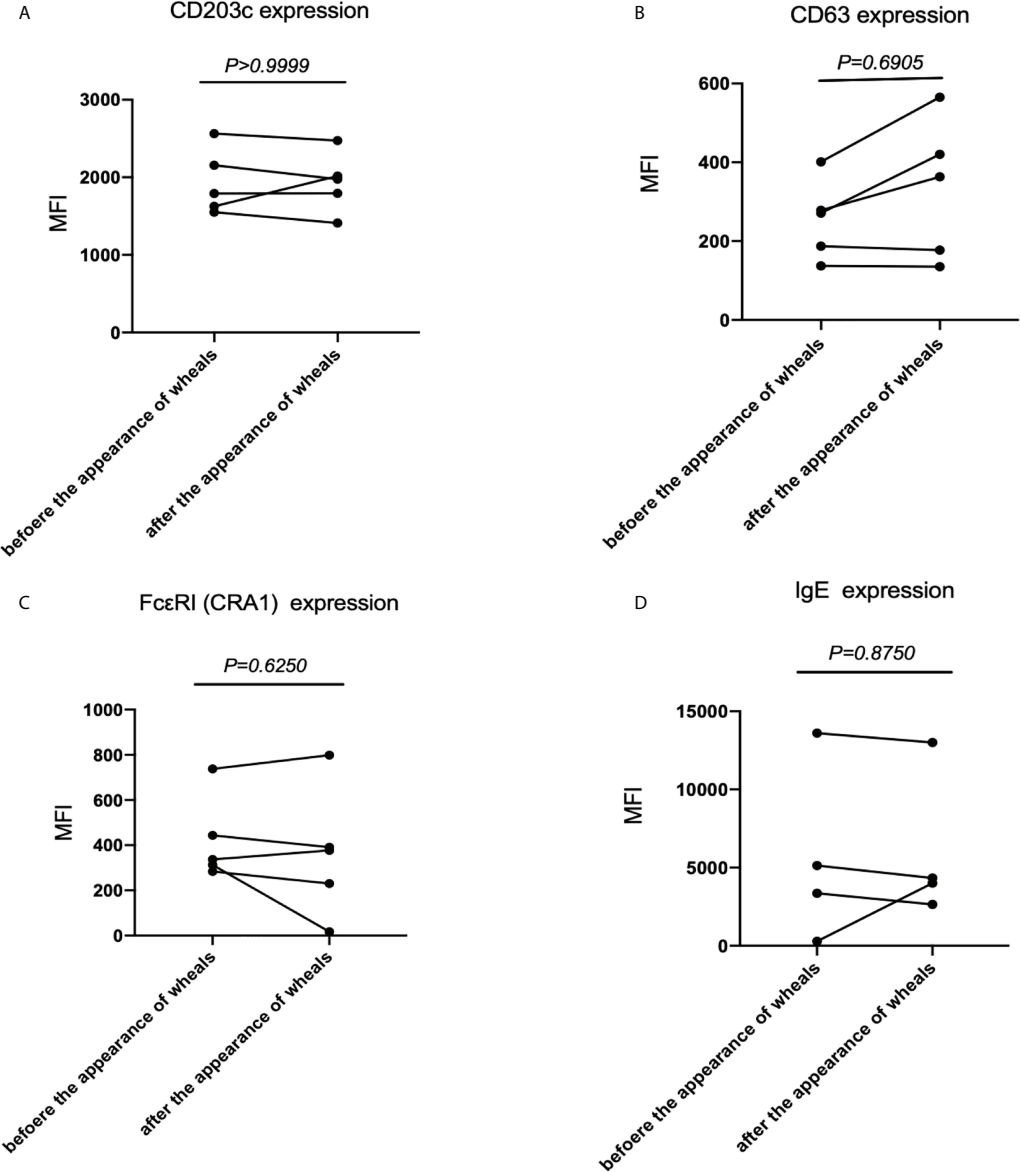
CD203c, CD63, IgE and FcϵRI levels at steady state before and after the appearance of wheals in patients with CholU as a subgroup of CIndU. Comparison of **(A)** CD203c expression on basophils, **(B)** CD63 expression on basophils, **(C)** FcϵRI expressions on basophils, and **(D)** IgE expressions on basophils before and after the appearance of wheals. Statistical analysis was performed by Wilcoxon test.

Thirdly, we examined CD203c, CD63, FcϵRI and IgE expression levels on basophils at steady state in patients with CholU before and after the appearance of wheals. There were no significant differences in the CD203 expression on basophils (**Figure 3A**), CD63 expression on basophils (**Figure 3B**), FcϵRI expression on basophils (**Figure 3C**) and in the levels of cell-bound IgE on basophils (**Figure 3D**) before and after the appearance of wheals.

### Measurement of CD203c and CD63 expressions after anti-IgE or FcϵRI stimulation of basophils in patients with CholU as a subgroup of CIndU before and after the appearance of wheals

**Figure 4:**
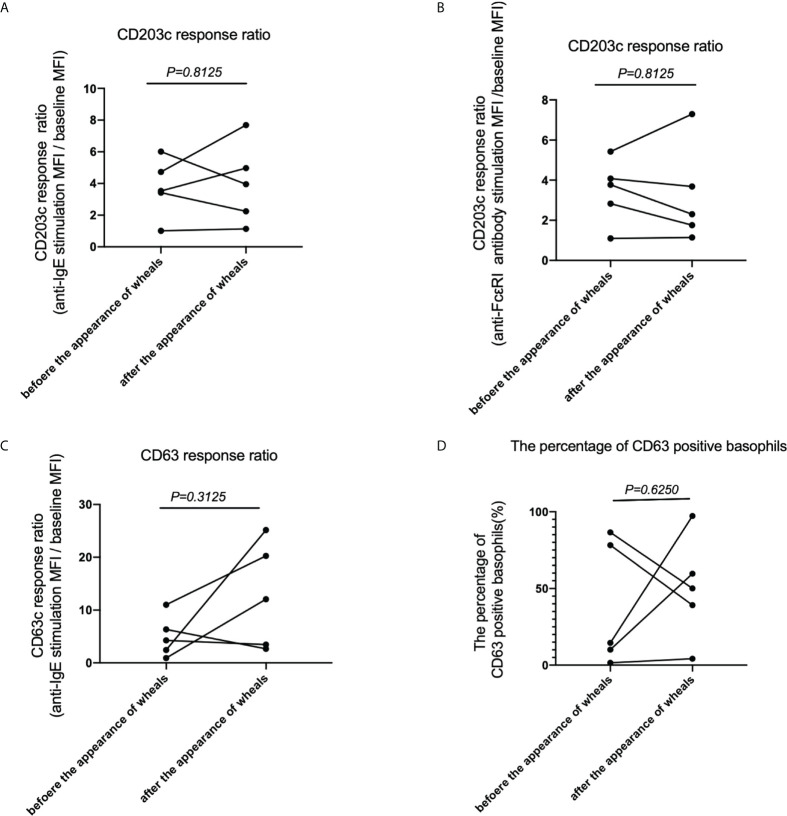
CD203c response ratio, CD63 response ratio and the percentage of CD63 positive basophils before and after the appearance of wheals in patients with CholU as a subgroup of CIndU. Comparison of CD203c response ratios of basophils when stimulated with **(A)** anti-IgE or **(B)** anti-FcϵRI antibody before and after the appearance of wheals. Comparison of **(C)** CD63 response ratios of basophils when stimulated with anti-IgE and **(D)** the percentage of CD63 positive basophils when stimulated with anti-IgE before and after the appearance of wheals. Statistical analysis was performed by Wilcoxon test.

Finally, we analyzed the expression of the activation markers CD203c and CD63 with anti-IgE or FcϵRI stimulation of basophils in patients with CholU before and after the appearance of wheals to examine basophil reactivity *via* FcϵRI. When peripheral blood basophils were stimulated with anti-IgE antibodies, the upregulation of CD203c expression on basophils after wheals appeared were equivalent to that before wheals appeared (**Figure 4A**). When peripheral blood basophils were stimulated with anti-FcϵRI, the upregulation of CD203c expression on basophils after wheals appeared was equivalent to that before wheals appeared (**Figure 4B**). When peripheral blood basophils were stimulated with anti-IgE antibodies, similar results were obtained when the detection activation marker was also CD63 (**Figure 4C**). The percentage of CD63 positive basophil also showed similar results when peripheral blood basophils are stimulated with anti-IgE antibody before and after the appearance of wheals. (**Figure 4D**).

## Discussion

In this study, we focused on the characteristics related to the steady state of basophils, FcϵRI-mediated responsiveness, and expression of IgE-related molecules in patients with CIndU. The degree of basophil activation at steady state in patients with CIndU was higher than in HCs. And then basophil responsiveness *via* FcϵRI stimulation with anti-IgE or anti-FcϵRI antibody in patients with CIndU was equivalent to that with HCs, and higher than that with CSU. In addition, no abnormalities were observed for the IgE and FcϵRI expressions on the surface of basophils in patients with CIndU. In addition, When we induced wheals in patients with CholU and performed a BAT before and after the appearance of wheals, no significant changes were found.

Basophils in patients with severe CSU might be mildly activated by autoantigens or autoantibody-related IgE pathways in the blood and persistently release small amounts of histamine (16). As a result, basophils in patients with CSU were exhausted and their responsiveness *via* FcϵRI was low (6) (7). In contrast, this study revealed that steady-state basophils in patients with CIndU had higher CD203c and CD63 than HCs, but there were no abnormalities in the responsiveness of basophils to stimulation with anti-IgE or FcϵRI antibodies. These findings indicate that basophils at steady state in CIndU patients may be weakly self-activated by unknown mechanism, whereas the basophil responsiveness in CIndU patients is not abnormal. In CholU as a subgroup of CIndU, FcϵRI-mediated responsiveness of peripheral blood basophils and expression of FcϵRI and IgE did not change significantly before and after the appearance of the wheals.This FcϵRI-mediated responsiveness of basophils and absence of abnormalities related to surface markers in CholU as a representative of CIndU may indicate a minor role of basophils in the pathogenesis in CIndU compared with CSU. It makes sense that basophils, which are mainly present in blood vessels, play a minor role in CIndU. This can be because sweat that leaks into the dermis from sweat ducts in CholU and serum-derived factors that are changed by sunlight reaching the dermis in solar urticaria are highly likely to act as allergens that induce urticaria in the dermis, respectively.

Our result regarding the expressions of FcϵRI and IgE on peripheral blood basophils is different from a previously reported result (11). This difference might be related to the high proportion of patients with CholU in our study. The statistical differences between CSU and CIndU in the presence of total IgE and baseline angioedema, and the history of atopy, might be associated with our high proportion of CIndU patients with CholU. In addition, there is a significant difference in the disease duration between CIndU and CSU. Differences in disease duration affected the responsiveness of basophils in patients with CSU (6), but no abnormalities in the responsiveness of basophils were observed in patients with CIndU, regardless of the short or long disease duration. Therefore, we believe that the difference in disease duration between CSU and CIndU does not affect the difference in basophil responsiveness between the two. The significantly higher expression of CD203c and CD63 on basophils at steady state in CIndU patients compared to HCs may also be influenced to the higher proportion of CholU complicated by AD. Indeed, we previously reported higher CD203c and CD63 expression on basophils at steady state in AD patients (17). Therefore, a population that does not differ statistically should be analyzed. There were several study limitations including the small number of cases and low diversity of disease subtypes in CIndU. In future studies, a higher number of cases should be enrolled to confirm the role of basophils and refine therapeutic targets for CIndU.

## Data availability statement

The original contributions presented in the study are included in the article/**Supplementary Materials**, further inquiries can be directed to the corresponding author.

## Ethics statement

The studies involving human participants were reviewed and approved by The Institutional Review Board of Kobe University. The patients/participants provided their written informed consent to participate in this study.

## Author contributions

MM and AF conceived the idea of the study. MM, YO, and SI developed the statistical analysis plan and conducted statistical analyses. MM and AF contributed to the interpretation of the results. AF, KW, and TF supervised the conduct of this study. All authors reviewed the manuscript draft and revised it critically for intellectual content. All authors approved the final version of the manuscript to be published.

## Funding

This work was supported in part by a Grant-in-Aid for Scientific Research (C) and a Grant-in-Aid for Young Scientists (B) (JSPS KAKENHI Grant Numbers 20K08651 and 19K19722) from the Ministry of Education, Culture, Sports, Science and Technology, Japan (to AF and KW).

## Acknowledgments

We thank J. Ludovic Croxford, PhD, from Edanz (https://jp.edanz.com/ac) for editing a draft of this manuscript.

## Conflict of interest

The authors declare that the research was conducted in the absence of any commercial or financial relationships that could be construed as a potential conflict of interest.

## Publisher’s note

All claims expressed in this article are solely those of the authors and do not necessarily represent those of their affiliated organizations, or those of the publisher, the editors and the reviewers. Any product that may be evaluated in this article, or claim that may be made by its manufacturer, is not guaranteed or endorsed by the publisher.

## Supplementary material

Figure S1 Flow cytometry data analysis. On the FSC/SSC plot **(A)**, the basophil scatter gate and leukocyte gate are defined. On the CD3-PE-Cy7/SSC plot **(B)**, the CD3 negative population is defined. On the CRTH2-FITC/CD203c-PE plot **(C)**, both CRTH and CD203c positive groups are defined as basophils. The gating basophils on the CRTH2-FITC/CD203c-PE plot are non-activated basophils **(C)** and the gating basophils on the CRTH2-FITC/CD203c-PE plot are activated basophils with anti-IgE **(D)** The gating basophils on the CRTH2-FITC/CD63-Pacific Blue are non-activated basophils **(E)** and the gating basophils on the CRTH2-FITC/CD63-Pacific Blue are activated basophils with anti-IgE **(F)** FITC, Fluorescein isothiocyanate; FSC, forward scatter; PE, phycoerythrin; PE-Cy7, PE-cyanine 7; SSC, side scatter.

## Abbreviations

CIndU, Chronic inducible urticaria; CSU, Chronic spontaneous urticaria; FcϵRI, High-affinity IgE receptor; HCs, Healthy controls; BAT, Basophil activation test; CholU, Cholinergic urticaria; UCT, Urticaria control test.
