# Supplementary figures and images for: IgE receptor responsiveness of basophils in chronic inducible urticaria
Source: Front Immunol. 2022 Sep 23;13:995596. doi: 10.3389/fimmu.2022.995596 (PMC9539802; doi:10.3389/fimmu.2022.995596)

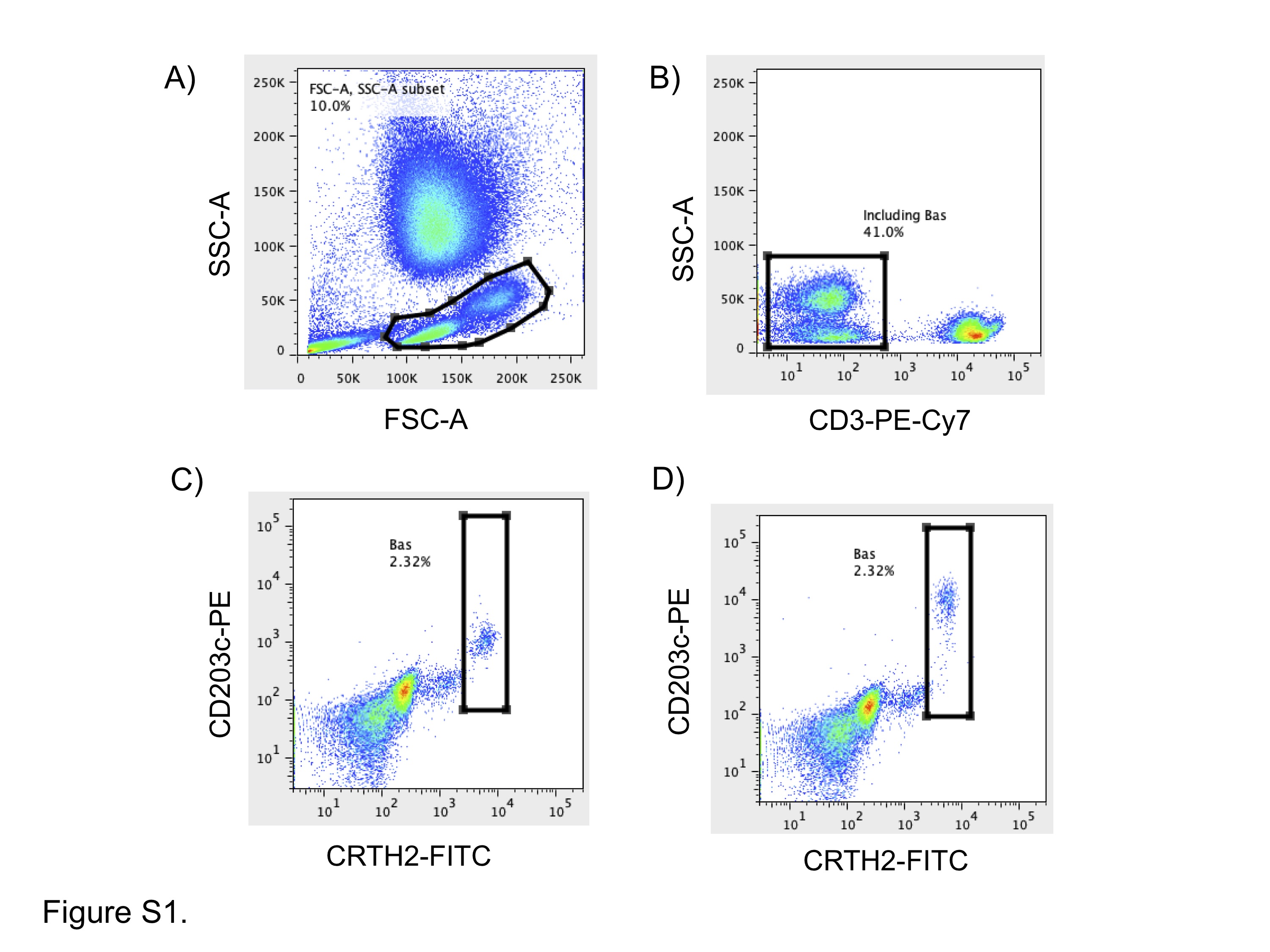

Supplement: Figure S1 — Flow cytometry data analysis. On the FSC/SSC plot (A), the basophil scatter gate and leukocyte gate are defined. On the CD3-PE-Cy7/SSC plot (B), the CD3 negative population is defined. On the CRTH2-FITC/CD203c-PE plot (C), both CRTH and CD203c positive groups are defined as basophils. The gating basophils on the CRTH2-FITC/CD203c-PE plot are non-activated basophils (C) and the gating basophils on the CRTH2-FITC/CD203c-PE plot are activated basophils with anti-IgE (D) The gating basophils on the CRTH2-FITC/CD63-Pacific Blue are non-activated basophils (E) and the gating basophils on the CRTH2-FITC/CD63-Pacific Blue are activated basophils with anti-IgE (F) FITC, Fluorescein isothiocyanate; FSC, forward scatter; PE, phycoerythrin; PE-Cy7, PE-cyanine 7; SSC, side scatter. [file Image_1.jpeg]

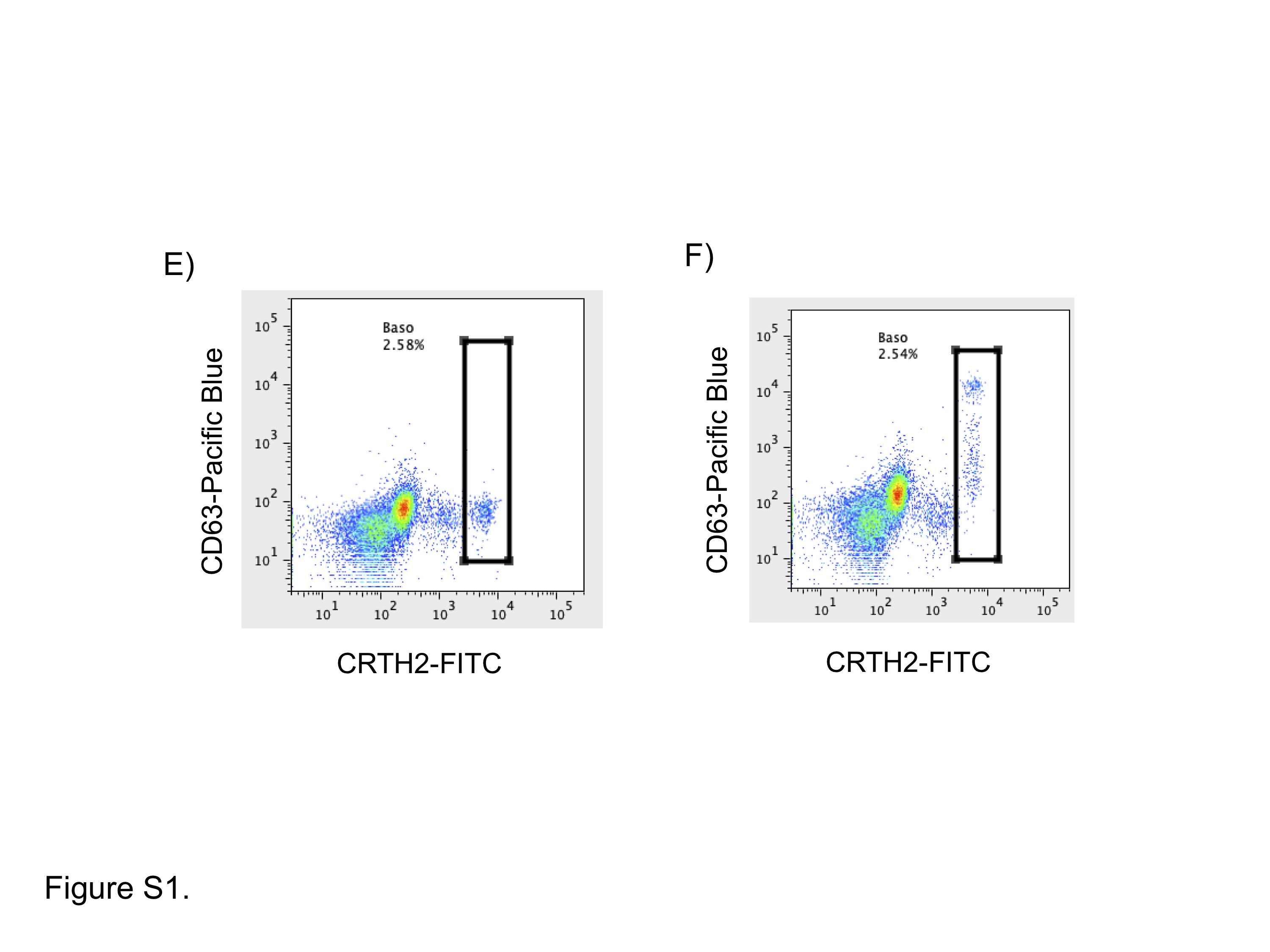

Supplement: Supplementary file 2 [file Image_2.jpeg]
